# Nesfatin-1 Promotes the Osteogenic Differentiation of Tendon-Derived Stem Cells and the Pathogenesis of Heterotopic Ossification in Rat Tendons via the mTOR Pathway

**DOI:** 10.3389/fcell.2020.547342

**Published:** 2020-12-03

**Authors:** Kai Xu, Zhanfeng Zhang, Mengyao Chen, Safwat Adel Abdo Moqbel, Yuzhe He, Chiyuan Ma, Lifeng Jiang, Yan Xiong, Lidong Wu

**Affiliations:** ^1^Department of Orthopedic Surgery, The Second Affiliated Hospital, School of Medicine, Zhejiang University, Hangzhou, China; ^2^Department of Orthopedic Surgery, The First People’s Hospital of Huzhou, Huzhou, China; ^3^Department of Medical Oncology, The Second Affiliated Hospital, Zhejiang University School of Medicine, Hangzhou, China

**Keywords:** nesfatin-1, tendinopathy, tendon-derived stem cells, osteogenic tendon differentiation, mTOR pathway

## Abstract

Heterotopic ossification (HO) is a pathological condition involved in tendinopathy. Adipokines are known to play a key role in HO of tendinopathy. Nesfatin-1, an 82-amino acid adipokine is closely reportedly associated with diabetes mellitus (DM), which, in turn, is closely related to tendinopathy. In the present study, we aimed to investigate the effects of nesfatin-1 on the osteogenic differentiation of tendon-derived stem cells (TDSCs) and the pathogenesis of tendinopathy in rats. *In vitro*, TDSCs were incubated in osteogenic induction medium for 14 days with different nesfatin-1 concentration. *In vivo*, Sprague Dawley rats underwent Achilles tenotomy to evaluate the effect of nesfatin-1 on tendinopathy. Our results showed that the expression of nesfatin-1 expression in tendinopathy patients was significantly higher than that in healthy subjects. Nesfatin-1 affected the cytoskeleton and reduced the migration ability of TDSCs *in vitro*. Furthermore, nesfatin-1 inhibited the expression of *Scx*, *Mkx*, and *Tnmd* and promoted the expression of osteogenic genes, such as *COL1a1*, *ALP*, and RUNX2; these results suggested that nesfatin-1 inhibits cell migration, adversely impacts tendon phenotype, promotes osteogenic differentiation of TDSCs and the pathogenesis of HO in rat tendons. Moreover, we observed that nesfatin-1 suppressed autophagy and activated the mammalian target of rapamycin (mTOR) pathway both *in vitro* and *in vivo*. The suppression of the mTOR pathway alleviated nesfatin-1-induced HO development in rat tendons. Thus, nesfatin-1 promotes the osteogenic differentiation of TDSC and the pathogenesis of HO in rat tendons via the mTOR pathway; these findings highlight a new potential therapeutic target for tendinopathy.

## Introduction

Tendinopathy is a progressive disorder of the tendon, accounting for over 30% of all musculoskeletal consultations (Andarawis-Puri et al., 2015). Tendinopathy is characterized by an increase in tendon thickness, stiffness, and tendon reinjury and a decrease in motor function (Burner et al., 2012; Rothan et al., 2013). Heterotopic ossification (HO) is a pathological condition that involves the formation of ectopic bone and often occurs during tendinopathy (Longo et al., 2018). Several risk factors are known to result in heterotopic tendon ossification, including obesity, age, sex, tendon vascularity, and gastrocnemius or soleus dysfunction (Longo et al., 2018). However, the mechanism underlying HO in tendinopathy remains unknown.

Tendon-derived stem cells (TDSCs) are isolated from tendon tissues and can self-renew, undergo multipotential differentiation, and maintain tendon homeostasis (Zhang et al., 2014; Jiang et al., 2018; Xu et al., 2020). Additionally, the osteogenic differentiation of TDSCs may account for tendon dysfunction and exacerbate the pathogenesis of tendinopathy.

Recently, numerous studies have shown that adipokines play a role in the pathogenesis of tendinopathy (Rothan et al., 2013; Jiang et al., 2018; Bauer et al., 2019). Adipokines, such as leptin accelerate the pathogenesis of HO in tendons. Nesfatin-1, an 82-amino acid adipokine derived from the prohormone nucleobindin-2 (NUCB2), was first identified in hypothalamic nuclei and is considered a key integrator in the regulation of food intake and energy balance (Li et al., 2012; Wu et al., 2014). Various studies have demonstrated that nesfatin-1 is closely related to diabetes mellitus (DM), during which the incidence of heterotopic tendon ossification is significantly higher than that in non-DM subjects of the same age (Aydeniz et al., 2008; Li et al., 2010; de Oliveira et al., 2011; Aslan et al., 2012). In early stage type 2 diabetes patients, the plasma nesfatin-1 levels were increased when compared with those in healthy controls, and reportedly decreased in type 2 diabetes patients receiving antidiabetic treatment (Zhai et al., 2017). However, few studies have focused on the effects of nesfatin-1 on the osteogenic differentiation of TDSCs and the pathogenesis of HO in tendons. Furthermore, the underlying intracellular molecular mechanisms downstream of nesfatin-1 have not been examined.

In the present study, we evaluated the expression of NUCB2 in human TDSCs and investigated the effects of nesfatin-1 on osteogenic TDSCs differentiation and pathogenesis of HO in rat tendons. Additionally, we investigated the potential mechanisms involved in these processes.

## Materials and Methods

### Reagents

Recombinant human nesfatin-1 and rapamycin (autophagy agonist) were purchased from Sigma-Aldrich (St Louis, MO, United States). Dulbecco’s modified Eagle’s medium (DMEM), penicillin/streptomycin, fetal bovine serum (FBS), and 0.25% trypsin were obtained from Gibco BRL (Grand Island, NY, United States).

### Patients

All patients were recruited by the Department of Orthopedic Surgery, the Second Affiliated Hospital, and signed informed consent forms approved by the Ethics Committee of the Second Affiliated Hospital, School of Medicine, Zhejiang University, Hangzhou, China. The tendons in the tendinopathy group (TD) were obtained from 17 patients (7 females and 10 males; aged 34 to 57 years old) with torn rotator cuffs who underwent reparative surgery, with a mean tear size of 2.1 cm^2^. Patients were only included if clinically detectable evidence of tendinopathy was detected on the preoperative MRI scan. An independent negative control group (NC) was composed of 17 patients (6 females and 11 males; aged 31 to 63 years old) who underwent anterior cruciate ligament autografts with no clinically detectable evidence of tendinopathy on a preoperative MRI scan. There was no difference between clinical characteristics, including age, body mass index, sex, diabetes, hypertension, and peripheral artery disease. All the tendon tissues were treated were frozen using liquid nitrogen and then ground. Total RNA was extracted using the TRIzol^TM^ Plus RNA Purification Kit (Invitrogen, Carlsbad, CA, United States). Nesfatin-1 expression in the human tendon tissue was evaluated by quantitative real-time PCR analysis.

### Cell Culture and Osteogenic Differentiation

This study was approved by the Institutional Animal Care and Use Committee of Zhejiang University (Hangzhou, China). After euthanasia, Achilles tendons were obtained from 3-week-old Sprague Dawley rats (Zhejiang Academy of Medical Sciences Hangzhou, China). The tissue was minced and incubated with type I collagenase (3 mg/mL) on a horizontal shaker at 37°C for 3 h to isolate the tenocytes. The tendon-derived cells were resuspended into single-cell suspensions and plated in DMEM supplemented with 10% FBS, 100 units/mL penicillin, and 100 μg/mL streptomycin for 7 days. The colonies were collected at passage 0 (P0). The cells were used at P3 (Xu et al., 2020). For osteogenic differentiation, the cells were incubated in osteogenic induction medium (Cyagen Biosciences, Suzhou, China) for 14 days. Nesfatin-1 (0.1, 1, or 10 ng/mL) was added to the osteogenic medium in the presence or absence of rapamycin (10 nM) (Jiang et al., 2018), the controls were cultured with an equal volume of DMSO in the osteogenic medium.

### Identification of Surface Markers

TDSCs were incubated with fluorescent primary antibodies on ice for 60 min, washed three times with phosphate-buffered saline (PBS), and analyzed by flow cytometry. The following fluorescent primary antibodies were used: FITC anti-rat CD29, FITC anti-rat CD44, PE anti-rat CD45, and PE anti-rat CD90 (BioLegend, San Diego, CA, United States). FITC isotype control and PE iso control were added to the NC samples (BioLegend, San Diego, CA, United States).

### Multipotency Analysis

TDSCs were incubated with specific differentiation media to analyze the cell multipotency. For osteogenic differentiation, TDSCs were incubated with osteogenic induction medium (Cyagen Biosciences, Suzhou, China) for 14 days, and Alizarin Red staining was performed to confirm osteogenic differentiation. For chondrogenic differentiation, chondrogenesis was performed in pellet culture with chondrogenic induction medium (Cyagen Biosciences, Suzhou, China) for 21 days, and Safranin O staining was used to confirm chondrogenic differentiation. For adipogenic differentiation, TDSCs were incubated in adipogenic induction and maintenance medium (Cyagen Biosciences, Suzhou, China) for 14 days, and Oil Red staining was performed to confirm the adipogenic differentiation.

### Cell Viability Analysis

The cytotoxicity of nesfatin-1 on TDSCs was assessed via the CCK-8 assay. TDSCs (1 × 10^4^ cells per well) were seeded in 96-well plates and treated with different concentrations of nesfatin-1 for 48 h (Lu et al., 2018). Cell viability was determined using the CCK-8 assay according to the manufacturer’s instructions.

### Scratch Assay

A scratch assay was used to evaluate the ability of TDSCs to migrate after treatment. TDSCs (1 × 10^5^ cells per well) were seeded in 6-well plates for 24 h. A straight line was cut into the cell layer with the tip of a P200 pipette. After washing with PBS, TDSCs were cultured with the corresponding treatment medium without serum. The gap between the two sides of the scratch was photographed after 0, 12, 24, and 48 h of incubation. The ratio of the open wounds was measured to evaluate the migration ability of TDSCs.

### Quantitative Real-time PCR (qRT-PCR) Analysis

The TRIzol^TM^ Plus RNA Purification Kit (Invitrogen) was used to extract the total RNA from TDSCs. After measuring and adjusting RNA concentrations using a nucleic acid detector, the total RNA was reverse transcribed into cDNA with the PrimeScript^TM^ RT Master Mix (TAKARA). Then, cDNA samples were amplified with SYBR^TM^ Premix Ex Taq^TM^ II (TAKARA) using an ABI StepOnePlus System. The primers used are shown in Table 1, and the expression of scleraxis *(Scx)*, mohawk homeobox *(Mkx)*, tenomodulin *(Tnmd)*, Collagen Type I Alpha 1 Chain *(Col1a1)*, alkaline phosphatase *(Alp)*, and runt-related transcription factor 2 *(RUNX2)* was detected. GAPDH was used as an endogenous control. All the assays were performed in triplicate, and data were calculated using the 2(−ΔΔCT) method.

**TABLE 1:** Primer sequences used in this study.

Gene	Forward	Reverse
Human GAPDH	CCATGACAACTTTGGTATCGTGGAA	GGCCATCACGCCACAGTTTC
Human NUCB2	CCCGGCCAGAACGTGTTA	CTTCGCACTTTCCACAGGGT
Rat GAPDH	GAAGGTCGGTGTGAACGGATTTG	CATGTAGACCATGTAGTTGAGGTCA
Rat Scx	AACACGGCCTTCACTGCGCTG	CAGTAGCACGTTGCCCAGGTG
Rat Mkx	TTTACAAGCACCGTGACAACCC	ACAGTGTTCTTCAGCCGTCGTC
Rat Tnmd	TGGGGGAGCAAACACTTCTG	TCTTCTTCTCGCCATTGCTGT
Rat ALP	ACCCTGCCTTACCAACTCATT	TCTCCAGCCGTGTCTCCTC
Rat Col1a1	CATCGGTGGTACTAAC	CTGGATCATATTGCACA
Rat RUNX2	CACA AGTGCGGTGCAAACTT	GCAGCCTTAAATATTACTGCATGG

### Western Blot Analysis

After the different treatments, TDSCs were washed twice and resuspended in PBS. Then, all samples were immediately lysed in RIPA lysis buffer containing protease and phosphatase inhibitors for 30 min to extract the proteins. A BCA protein assay kit (Beyotime Biotechnology, Shanghai, China) was obtained to quantify the protein concentrations. The samples were separated using sodium dodecyl sulfate-polyacrylamide gels and transferred into nitrocellulose membranes. After blocking with 5% BSA for 1 h, the membranes were cut into sections based on the different protein molecular weights. The different sections were incubated with the following monoclonal antibodies: Col1a1, Alp, GAPDH, LC3A/B, p62, p-mTOR, mTOR (Abcam, Cambridge, United Kingdom), RUNX2, p-S6, and S6 (Cell Signaling Technology, Danvers, MA, United States), at 4°C overnight. These sections were incubated with secondary antibodies for 2 h, luminesced by the Pierce^TM^ ECL western blotting substrate, and detected using the Bio-Rad ChemiDoc System. The ImageJ software was used to measure the gray values for all the western blot bands. All the assays were performed in triplicate.

### Phalloidin Staining and Quantitative Analysis of Fluorescent Images

Phalloidin staining (Yeasen Biotechnology, Shanghai, China) was used to detect the actin cytoskeleton in TDSCs. The cells (5 × 10^4^ cells per well) were seeded in 12-well plates and treated with different concentrations of nesfatin-1. After fixation with 4% paraformaldehyde for 10 min, the cells were incubated with a phalloidin solution according to the manufacturer’s instructions, and the fluorescence was visualized by confocal microscopy (Carl Zeiss, Oberkochen, Germany).

For TDSCs morphology, changes were assessed using the average cell surface area and aspect ratio (major cell axis/minor cell axis) with the ImageJ software. The anisotropy of actin fibers was measured using OrientationJ plug-in for ImageJ to evaluate the arrangement of stress fibers.

### Alkaline Phosphatase (ALP) Staining

TDSCs were differentiated into osteoblasts with osteogenic induction medium. Briefly, 5 × 10^4^ TDSCs were seeded in 12-well plates. After reaching 80% confluence, the cells were cultured with osteogenic induction medium with different nesfatin-1 concentrations for 6 days. The cells were fixed with 4% paraformaldehyde for 30 min. Then, the cells were stained using the Alkaline Phosphatase Color Development Kit (Beyotime).

### Alizarin Red Staining

Alizarin Red staining was used to evaluate the mineral deposition induced by osteogenic differentiation in TDSCs. Briefly, 5 × 10^4^ TDSCs were seeded in 12-well plates. After reaching 80% confluence, the cells were cultured with osteogenic induction medium with different nesfatin-1 concentrations for 2 weeks. Then, the cells were fixed with 4% paraformaldehyde for 30 min. Finally, the cells were stained with a 0.1% solution of Alizarin Red (Sigma-Aldrich) for 10 min.

### Animal Model

Six-week-old male Sprague Dawley rats (*n* = 60, 200–250 g) were purchased from Zhejiang Academy of Medical Sciences (Hangzhou, Zhejiang, China). The rats were randomly divided into four groups: HO, HO + RA, HO + NES, and HO + NES + RA. All rats underwent Achilles tenotomy (Xu et al., 2020). The rats were anesthetized using pentobarbital (40 mg/kg), and a bilateral midpoint Achilles tenotomy was performed through a posterior approach under aseptic conditions. A week after the Achilles tenotomy, all groups were treated differently. In the HO + RA + NES group (*n* = 15) and HO + NES group (*n* = 15), the rats were injected with 0.1 mL of 10 ng/mL nesfatin-1 (NES) once per week in the region surrounding the Achilles tendon. The other two groups were injected with 0.1 mL PBS once per week as a control. In the HO + RA group (*n* = 15) and HO + NES + RA group (*n* = 15), the rats were intraperitoneally administered rapamycin (RA) (1 mg/kg/day) daily. The other two groups were intraperitoneally administered the saline vehicle as a control daily. During the treatment period, all rats were allowed free cage activities. Eight weeks after Achilles tenotomy, the rats were euthanized, and their Achilles tendon tissues were harvested for further study. This study was conducted in accordance with the NIH guidelines (NIH Pub No 85-23, revised 1996), and the protocol was approved by the Ethics Committee of the Second Affiliated Hospital, School of Medicine, Zhejiang University, Hangzhou, China.

### Micro-CT Analyses

Following the animal experiment, all rats were euthanized, and their right hind legs were collected for micro-computed tomography analyses (μCT 100, SCANCO Medical AG, Wangen-Brüttisellen, Zurich, Switzerland). The parameter settings were as follows: 90 kV, 200 μA, and 30-μm slice thickness. The newly formed bone volume (BV, mm^3^) following HO remodeling of the Achilles tendons was calculated and quantified using SCANCO Medical software.

### Histological Analysis

Tendon samples were embedded in paraffin and continuously cut into 5-μm sections. These sections were stained with HE staining, Safranin O staining, and modified Masson staining according to the manufacturer’s instructions.

### Immunofluorescence

Tendon sections were incubated with the primary antibody at 4°C overnight. Then, the sections were incubated with a secondary antibody conjugated to fluorescein isothiocyanate (FITC). The nuclei were stained with DAPI according to the manufacturer’s instructions. The results were visualized by fluorescence microscopy.

### Immunohistochemistry

Tendon sections were rehydrated and incubated with 3% hydrogen peroxide for 30 min at room temperature. Then, 5% BSA was used to block these sections for 2 h. Then, the sections were incubated with primary antibodies against nesfatin-1 (USCN, Wuhan, China) and Tnmd (USCN, Wuhan, China) at 4°C overnight. After interaction with the HRP-conjugated secondary antibodies, the sections were visualized with DAB, and nuclei were stained with hematoxylin. The results were visualized using optical microscopy.

### Statistical Analyses

All the assays were performed in triplicate. Data are presented as means ± standard deviation (SD). The Student’s *t*-test was performed to assess statistically significant differences between the results of two experimental groups. For multiple comparisons, one-way analysis of variance (ANOVA) with Tukey’s *post hoc* test was performed. A *p*-value < 0.05 was considered to indicate statically significant differences.

## Results

### Isolation and Identification of TDSCs

**FIGURE 1:**
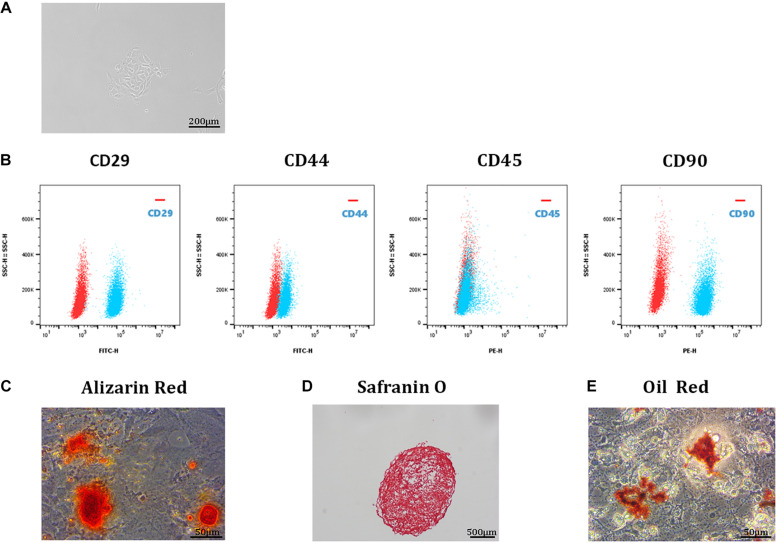
Isolation and identification of TDSCs. **(A)** A single-cell suspension of 1 cell/μL was prepared and cultured in a 12-well plate at 37°C with 5% CO_2_. These TDSCs were able to form small colonies and were photographed at 4 day. Scale bar, 200 μm. **(B)** Representative flow cytometric profiles of TDSCs stained for the surface markers CD29, CD44, CD45, and CD90 (red: control; blue: fluorescent antibody). Scale bar, 500 μm. **(C)** Alizarin Red staining of TDSCs osteogenic differentiation. Scale bar, 50 μm. **(D)** Safranin O staining of TDSCs chondrogenic differentiation. Scale bar, 500 μm. **(E)** Oil Red staining of TDSCs adipogenic differentiation. Scale bar, 50 μm.

Single-cell suspensions of tendon-derived cells were cultured in DMEM. These round cells were able to form small colonies (Figure 1A). Cell surface marker analysis was used to identify the stem status of these clonogenic cells. The results showed that these cells expressed high levels of stem cell markers (CD29, CD44, and CD90) and undetectable levels of a leucocyte marker (CD45) (Figure 1B; Ogata et al., 2018). In addition, the multipotency analysis of TDSCs revealed that these cells exhibited stem cell properties. Alizarin Red staining of osteogenic cultures showed calcium deposits within the cell monolayer (Figure 1C); Safranin O staining of chondrogenic pellet cultures presented the potency of trans-differentiation into chondro-lineage (Figure 1D); Oil Red staining confirmed the potency of adipogenic differentiation (Figure 1E).

### Cell Viability Assay

**FIGURE 2:**
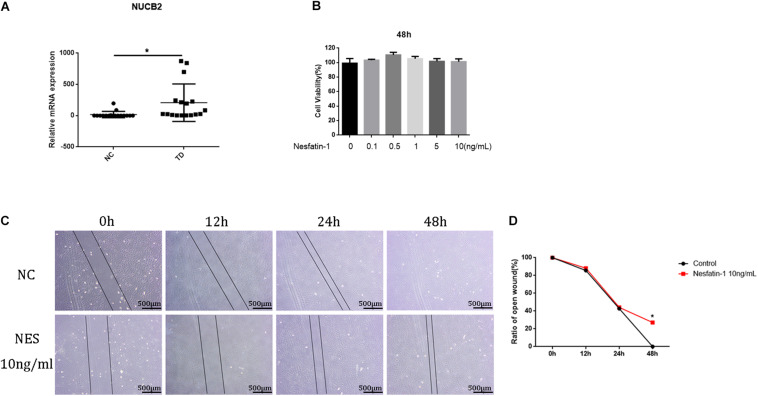
Cell viability assay. The expression of NUCB in human tendon tissues was detected by qRT-PCR. **(A)** The TDSCs were treated with increasing concentrations of Nesfatin-1 (0, 0.1, 0.5, 1.5, or 10 ng/mL) for 48 h. **(B)** The effects of Nesfatin-1 on rat TDSCs viability were evaluated by the CCK-8 assay. **(C,D)** a scratch assay was used to evaluate the effects of Nesfatin-1 on the ability of TDSCs to migrate. Scale bar, 500 μm. *p < 0.05.

qRT-PCR was used to investigate the expression of NUCB2 in human tendon tissues. The results showed that the NUCB2 levels were higher in tendinopathy patients than in healthy controls (Figure 2A). To investigate the effect of nesfatin-1 on TDSC viability, a CCK-8 assay was used. The results revealed that nesfatin-1 had no adverse effect on TDSC viability, within the range from 0 to 10 ng/mL at 48 h (Figure 2B). The scratch assay showed that 10 ng/mL nesfatin-1 reduced TDSC migration (Figures 2C,D). These findings indicated that nesfatin-1 may influence the migration of TDSCs.

### Effect of Nesfatin-1 on TDSCs Phenotype Maintenance and Cytoskeleton

**FIGURE 3:**
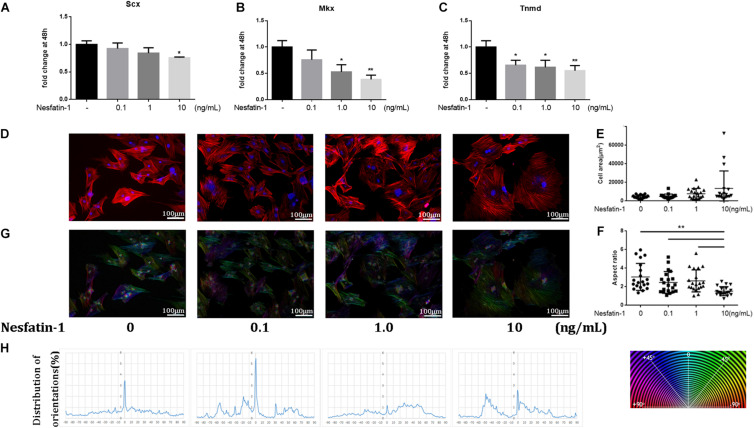
Effect of Nesfatin-1 on TDSCs phenotype maintenance and cytoskeleton. **(A–C)** TDSCs were treated with different concentrations of Nesfatin-1 for 48 h. The mRNA levels of SCX, Mkx, and Tnmd were evaluated via qRT-PCR. **(D)** The TDSCs were treated with different concentrations of Nesfatin-1 for 24 h. The actin cytoskeleton organization was analyzed by phalloidin staining. F-actin stained in red and cell nuclei in blue. **(E,F)** Quantitative analysis of cell surface area and aspect ratio (major cell axis/minor cell axis) with ImageJ software. **(G,H)** OrientationJ plug-in for ImageJ was used to analysis the arrangement of stress fibers. The values are expressed as the mean ± standard deviation (SD). *p < 0.05, **p < 0.01 vs. control group. Scale bar, 100 μm.

The effect of nesfatin-1 on TDSC phenotype maintenance was detected using tenogenic markers, such as *Scx, Mkx*, and *Tnmd*. The expression of these markers was detected via qRT-PCR. The results showed that nesfatin-1 significantly inhibited the expression of *SCX, Mkx*, and *Tnmd* (Figures 3A–C), which demonstrated that nesfatin-1 may affect TDSC phenotype maintenance. The cytoskeleton is linked to cell migration and the TDSC phenotype (Wang et al., 2015; Tang and Gerlach, 2017). To study the effects of nesfatin-1 on the in TDSC cytoskeleton, filamentous actin (F-actin) was visualized by phalloidin staining (Figure 3D). The F-actin arrangement was mainly organized into parallel stress fibers that stretched normally along the major axis. The results showed that exposure to nesfatin-1 revealed no major changes in cell area (Figure 3E). However, a significant decrease in the aspect ratio was observed for TDSCs cultured in 10 ng/mL nesfatin-1, when compared with other concentrations (Figure 3F). As shown in Figure 3, the morphology of the TDSCs, which usually exhibits a fibroblast-like shape, changed into a polygon-like shape following nesfatin-1 stimulation. OrientationJ plug-in for ImageJ was used to analyze the arrangement of stress fibers. The result showed that compared with lower concentration groups (0 and 0.1 ng/mL), higher concentration groups (1.0 and 10 ng/mL) performed random disposition, which translated into the absence of a peak in the corresponding orientation plot. These results revealed that nesfatin-1 increased the anisotropy of F-actin, changed the cellular cytoskeleton of TDSCs, and might influence the migration of TDSCs.

### Nesfatin-1 Promotes the Osteogenic Differentiation of TDSCs *in vitro*

**FIGURE 4:**
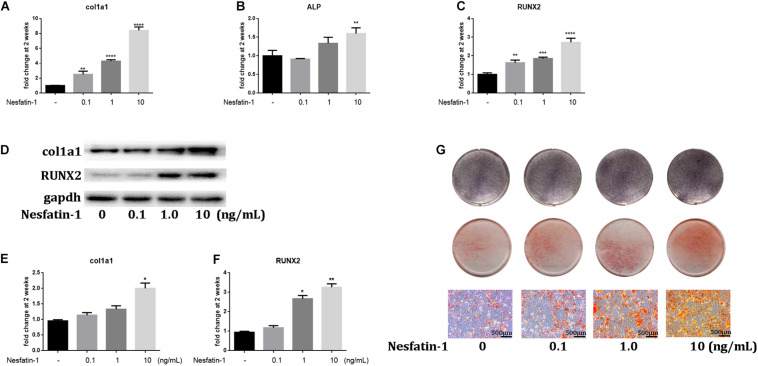
Nesfatin-1 promotes the osteogenic differentiation of TDSCs *in vitro.* The TDSCs were cultured in osteogenic induction medium and treated with different concentrations of Nesfatin-1. **(A–C)** The expression of col1a1, ALP, and RUNX2 was evaluated via qRT-PCR after the cells were cultured for 14 days. **(D–F)** The expression of col1a1 and RUNX2 was evaluated via Western blot after the cells were cultured for 14 days. **(G)** ALP staining was performed in cells cultured for 6 days. Alizarin Red staining was performed in cells cultured for 14 days. The values are expressed as the mean ± standard deviation (SD). *p < 0.05, **p < 0.01, ***p < 0.001, ****p < 0.0001 vs. control group. Scale bar, 500 μm.

HO is a pathologic condition in tendinopathy. To investigate the effect of nesfatin-1 on HO in tendinopathy, we detected the expression of osteogenic markers such as COL1A1 (Valles et al., 2020), ALP (Gong et al., 2016), and RUNX2 (Berendsen and Olsen, 2015), under nesfatin-1 stimulation *in vitro*. The qRT-PCR results showed that the expression of COL1A1, ALP, and RUNX2 significantly increased as the nesfatin-1 concentration increased (Figures 4A–C). The protein expression levels of COL1A1 and RUNX2, as measured by western blotting, were similar to the mRNA expression levels measured by qRT-PCR (Figures 4D–F). Furthermore, ALP staining and Alizarin Red staining were performed and revealed that nesfatin-1 accelerated the osteogenic differentiation of TDSCs in a dose-dependent manner (Figure 4G).

### The Effect of Nesfatin-1 on Osteogenic Differentiation and Autophagy via the Mammalian Target of Rapamycin (mTOR) Pathway

**FIGURE 5:**
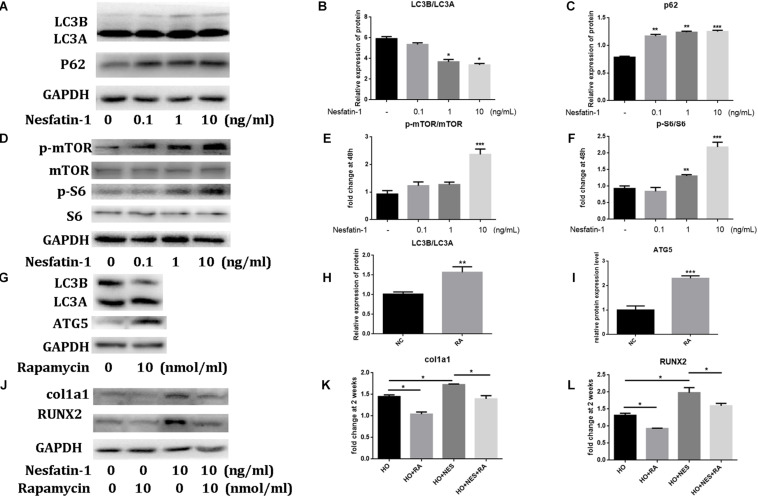
The effect of Nesfatin-1 on osteogenic differentiation and autophagy via the mTOR pathway. **(A–C)** The TDSCs were treated with different concentrations of Nesfatin-1 for 6 h. The expression levels of LC3A, LC3B, and p62 were evaluated via Western blot. **(D–F)** The TDSCs were treated with different concentrations of Nesfatin-1 in osteogenic induction medium for 14 days. The expression levels of p-mTOR, mTOR, p-S6, and S6 were evaluated via Western blot. **(G–I)** Rapamycin (10 nmol/mL) was used to inhibit the activation of the mTOR pathway and activate the autophagy. **(J–L)** The Nesfatin-1-induced osteogenic response can be inhibited by rapamycin. *p < 0.05, **p < 0.01, ***p < 0.001.

Recent studies have observed that the autophagic response participated in the pathogenesis of HO (Dai et al., 2013; Liu et al., 2013). In this study, we using western blotting, we observed that nesfatin-1 significantly reduced the ratio of LC3B/LC3A, which is a biomarker of autophagy (Mizushima and Yoshimori, 2007), and increased the expression of p62, which is associated with autophagic degradation (Lee et al., 2011), in TDSCs (Figures 5A–C). These results suggested that nesfatin-1 inhibited autophagy in TDSCs. Then, we investigated the mTOR pathway to explore the underlying mechanism of nesfatin-1. After nesfatin-1 treatment in the presence of osteogenic induction medium for 14 days, western blot results demonstrated that nesfatin-1 significantly increased the phosphorylation of mTOR and S6 in a dose-dependent manner (Figures 5D–F). These results suggested that nesfatin-1 activated the mTOR pathway during osteogenic differentiation of TDSCs. Furthermore, we inhibited the activation of the mTOR pathway by rapamycin (10 nmol/mL) and activated autophagy by increasing the ratio of LC3B/LC3A and ATG5 (Figures 5G–I). We observed that the nesfatin-1-induced enhancement of col1a1 and RUNX2 was significantly decreased (Figures 5J–L). These experimental results suggested that nesfatin-1 induced mTOR pathway activation and inhibited autophagy, which accelerated the osteogenic differentiation of TDSCs.

### Nesfatin-1 Accelerates the Pathogenesis of Heterotopic Ossification Through the mTOR Pathway *in vivo*

**FIGURE 6:**
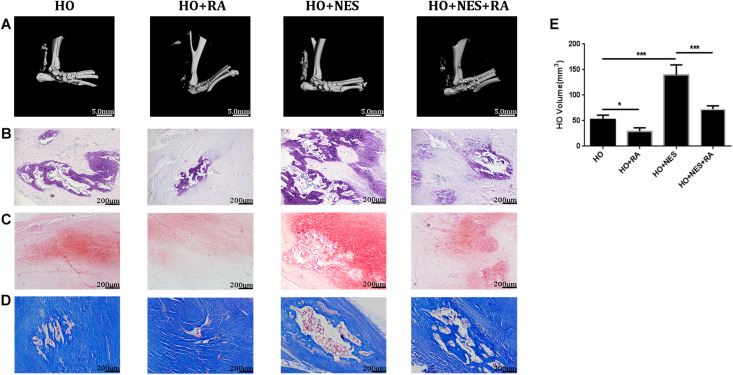
Nesfatin-1 accelerates the pathogenesis of heterotopic ossification *in vivo*. Sixty rats underwent Achilles tenotomy and were treated with Nesfatin-1 (10 ng/mL) and rapamycin (10 nmol/mL) for 8 weeks. Heterotopic bone formation was measured by micro-CT **(A,E)**. HE staining **(B)**, Safranin O staining **(C)** and modified Masson staining **(D)** were performed for histological analysis. The values are expressed as the mean ± standard deviation (SD). *p < 0.05, **p < 0.01 vs. the control group. Scale bar, 200 μm. ***p < 0.001.

**FIGURE 7:**
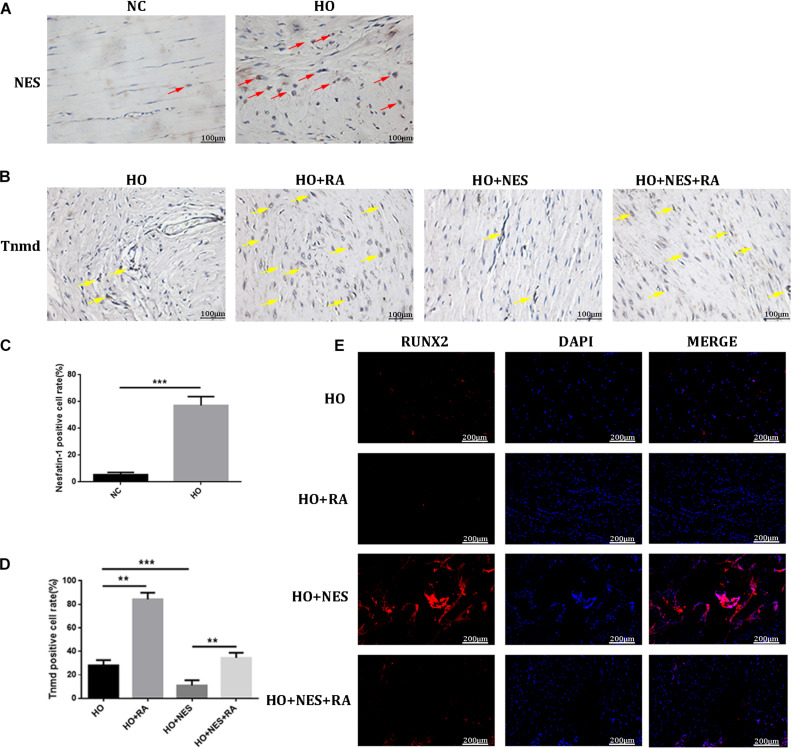
The effect of Nesfatin-1 on tendon healing *in vivo*. **(A–D)** The expression of Nesfatin-1 and Tnmd in the animal model was examined by IHC (red arrows indicated the positive cells of Nesfatin-1; yellow arrows indicated the positive cells of Tnmd), and the quantitative analysis of these proteins, Scale bar, 100 μm. **(E)** RUNX2 was a key indicator in HO and was detected by immunofluorescence, Scale bar, 200 μm. The values are expressed as the mean ± standard deviation (SD). *p < 0.05, **p < 0.01 vs. the control group. ***p < 0.001.

Micro-CT analyses were performed to measure the heterotopic bone formation of rat tendons induced by nesfatin-1. The results showed that new bone in the Achilles tendon was found in all rats. However, the size of these heterotopic bones differed. In the HO + NES group, the heterotopic bone was significantly larger than that in the HO group. In the HO + NES + RA group, the heterotopic bone was smaller than that in the HO + NES group but larger than that in the HO + RA group (Figures 6A,E). HE staining, Safranin O staining, and modified Masson staining were performed for histological analysis. The results showed that the arrangement of fibroblasts and collagen fibers was visibly disordered following nesfatin-1 treatment. In the HO + NES group, the calcified area was larger than in the HO group. Rapamycin reversed the increase in the calcified area induced by nesfatin-1 (Figures 6B–D). Based on immunohistochemistry, nesfatin-1 was increased in the HO group when compared with the normal control group (Figure 7A). Additionally, Tnmd was a key indicator of tendon healing and was detected by immunohistochemistry. The results showed that the Tnmd expression was low in the HO group and can be significantly rescued by rapamycin. Nesfatin-1 decreased the protein expression of Tnmd, which can also be significantly rescued by rapamycin (Figures 7B–D). Furthermore, we assessed the effect of nesfatin-1 on HO *in vivo* by evaluating RUNX2 expression. The immunofluorescence analysis showed that nesfatin-1 increased the protein expression of RUNX2, which could be rescued by rapamycin *in vivo* (Figure 7E).

**FIGURE 8:**
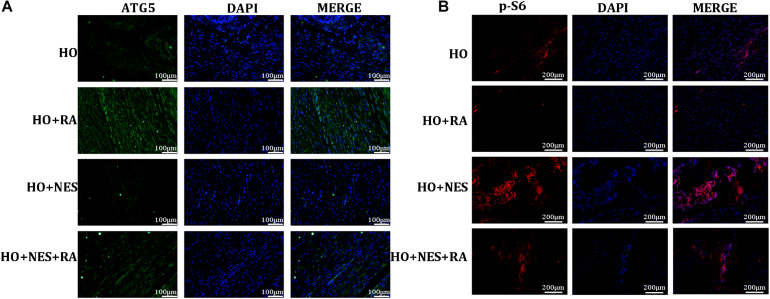
Nesfatin-1 promotes activation of the mTOR pathway *in vivo*. **(A)** The expression of ATG5 was evaluated as an indicator of autophagy by immunofluorescence, Scale bar, 100 μm. **(B)** p-S6 protein in the tissue sections was evaluated as an indicator of mTOR signaling by immunofluorescence, Scale bar, 200 μm. The values are expressed as the mean ± standard deviation (SD). *p < 0.05, **p < 0.01 vs. the control group.

As autophagy is deemed to play vital roles in tendon HO, we examined the effect of nesfatin-1 on tendon autophagy *in vivo*. As expected, rapamycin activated the expression of ATG5, a key indicator in autophagy. Nesfatin-1 suppressed ATG5 and this inhibition could be rescued by rapamycin (Figure 8A). Furthermore, we evaluated the activation of mTOR signaling by detecting the downstream marker of this pathway, namely p-S6, *in vivo*. We observed that nesfatin-1 could activate the expression of p-S6, and rapamycin undoubtedly reversed the activation of mTOR signaling (Figure 8B). The results described above suggested that nesfatin-1 accelerated the pathogenesis of HO through the mTOR pathway *in vivo*.

## Discussion

Chronic tendinopathy refers to a common, pathological condition in tendons, which often leads to chronic pain and tendon disability. The underlying pathogenesis of chronic tendinopathy remains poorly understood, and the main treatment involves symptom control. It is increasingly apparent that metabolic features play a significant role in tendinopathy (Castro et al., 2016). Furthermore, it is well-known that there are several different adipokines, including leptin, adiponectin, chemerin, and visfatin (Kershaw and Flier, 2004). Previous studies have suggested that adipokines are involved in the pathogenesis of tendinopathy. Rothan has reported that adiponectin, as an adipokine, improves TDSC proliferation and differentiation, suggesting that adiponectin is a potential therapeutic agent in diabetic tendinopathy (Rothan et al., 2013). Jiang has demonstrated that leptin, which is also an adipokine, promotes TDSC osteogenic differentiation and heterotopic bone formation (Jiang et al., 2018).

Nesfatin-1 is a secreted adipokine involved in feeding behaviors and body weight control. Reportedly, nesfatin-1 plays a regulatory role in patients with DM (Tekin and Cicek, 2019). DM has long been recognized as a well-known risk factor for the pathogenesis of tendinopathy (Abate et al., 2012). Nevertheless, no study has focused on the expression and effects of nesfatin-1 in tendinopathy. Our study first demonstrated that the NUCB2 levels in tendon tissues from tendinopathy patients were increased when compared with those from healthy controls. In the animal model of tendinopathy, the expression of nesfatin-1 was increased when compared with the NC. Furthermore, we investigated the mechanism of nesfatin-1 in the pathogenesis of tendinopathy.

In the present study, we observed that nesfatin-1 demonstrated no adverse effect on the TDSC viability, but reduced TDSC migration. Currently, evidence supports that TDSC migration is indispensable for tendon wound healing. During the tendon repair and regeneration process, TDSCs can migrate to the site of injury, differentiate into tenocytes, and replace the dysfunctional tenocytes involved in the pathophysiological process (Kohler et al., 2013). We observed that nesfatin-1 reduced the migration of TDSCs at 48h significantly when compared with the control group. Interestingly, previous studies have shown that the cytoskeleton plays an important role during cell migration (Hohmann and Dehghani, 2019). The regulation of the cytoskeleton is mediated by monomeric G-actin, filamentous F-actin, and actin-associated proteins (Rotty and Bear, 2014). To explore the mechanism of adverse effects on migration, we investigated the effects of nesfatin-1 on the cytoskeleton of TDSCs via phalloidin staining. The results showed that the anisotropy of F-actin induced by nesfatin-1 may change the cellular cytoskeleton. Base on alterations in the cellular cytoskeleton induced by nesfatin-1, we predicted that the ability of the cells to migrate was inhibited.

Besides self-renewal and migration, TDSCs can differentiate and maintain tendon homeostasis (Bi et al., 2007; Ni et al., 2012). In the pathological process of chronic tendinopathy, some abnormal matrix components (e.g., hypervascularity, acquisition of chondrocyte phenotypes, and calcification) are produced. Previous studies have suggested that the erroneous differentiation of TDSCs might result in depletion of the TDSC pool and ectopic chondro-ossification (Zhang and Wang, 2010). We observed that under the effect of nesfatin-1, TDSCs express lower tenogenic markers, including Scx, Mkx, and Tnmd. Notably, Scx, Mkx, and Tnmd are necessary for the maturation of collagen fibrils, and thus, these proteins are tenogenic markers. Scx, a basic helix-loop-helix (bHLH) transcription factor, is highly expressed in TDSCs (Yoshimoto et al., 2017). Murchison has reported that Scx−/− mutant mice suffer from severe tendon defects (Murchison et al., 2007). Mkx regulates type I collagen production and dramatically upregulates Scx by binding to the Tgfb2 promoter (Murchison et al., 2007). Liu observed that Mkx−/− mutant mice exhibited ectopic ossification in their Achilles tendons (Liu et al., 2019). Tnmd, a type II transmembrane glycoprotein, is predominantly expressed in tendon tissues (Dex et al., 2016). Docheva reported that Tnmd−/− mutant mice exhibit delayed maturation of collagen fibrils (Docheva et al., 2005). Our study revealed that nesfatin-1 significantly reduced the expression of the tenogenic markers, Scx, Mkx, and Tnmd, in a dose-dependent manner. Furthermore, our animal experiment verified that nesfatin-1 reduced the expression of Tnmd *in vivo*. These results suggested that nesfatin-1 may inhibit the production and maturation of collagen fibrils in TDSCs.

HO is widely considered a pathological characteristic of tendinopathy. Recent investigations have revealed that the osteogenic differentiation of TDSCs participates in the pathological process of tendon HO (Rothan et al., 2013; Jiang et al., 2018; Han et al., 2019). In the present study, we observed that nesfatin-1 increased osteogenic gene (*COL1A1, RUNX2*, and *ALP*) expression in TDSCs *in vitro* and promoted heterotopic bone formation in Achilles tendinitis *in vivo*. These results suggest that nesfatin-1 promotes the osteogenic differentiation of TDSCs and the pathogenesis of HO in rat tendons.

It has been reported that autophagy plays a crucial role in maintaining the self-renewal and stemness of TDSCs (Wu et al., 2011; Hudgens et al., 2016; Xu et al., 2020). In our study, we observed that nesfatin-1 reduced the ratio of LC3B/LC3A and increased the expression of p62, which suggested that Nesfatin-1 inhibited autophagy in TDSCs. In our previous study, we have reported that the mTOR pathway is involved in the pathological process of HO in TDSCs (Xu et al., 2020). Our data showed that nesfatin-1 significantly increased the phosphorylation of mTOR and S6 in a dose-dependent manner, indicating the role of mTOR pathway activation during osteogenic differentiation in TDSCs. To inhibit mTOR, we treated TDSCs with rapamycin, which inhibits mTORC1 (Cong et al., 2018). The western blot results showed that the nesfatin-1-induced osteogenic differentiation of TDSCs was significantly suppressed by rapamycin. *In vivo*, as expected, rapamycin suppressed heterotopic bone formation when compared with the HO group, which has been previously reported (Hino et al., 2018; Valer et al., 2019). Nesfatin-1 increased heterotopic bone formation and activation of mTOR signaling in the tendon, both decreased by rapamycin. Immunofluorescence and histological analysis both confirmed the consistent results described above. Therefore, the underlying mechanism of nesfatin-1 in the acceleration of TDSCs osteogenic differentiation and HO in rat tendons is associated with the activation of the mTOR pathway. Based on these result findings, we propose the therapeutic potential of targeting nesfatin-1 to combat tendinopathy in the future.

## Conclusion

Collectively, our data showed that nesfatin-1 inhibits cell migration, adversely affects tendon phenotypic maintenance, and promotes the osteogenic differentiation of TDSCs. Nesfatin-1 promotes the pathogenesis of HO in tendons via the mTOR pathway. These findings offer a potential therapeutic target for tendinopathy.

## Data Availability Statement

The datasets generated for this study are available on request to the corresponding author.

## Ethics Statement

The studies involving human participants were reviewed and approved by the Ethics Committee of The Second Affiliated Hospital, School of Medicine, Zhejiang University, Hangzhou, China. The patients/participants provided their written informed consent to participate in this study.

## Author Contributions

LJ, YX, and LW conceived and designed the study. KX, ZZ, MC, SM, and YH acquired, analyzed, and interpreted the data. ZZ drafted and edited the manuscript. All authors aided in revising this manuscript for intellectual content and approved the final version to be published.

## Conflict of Interest

The authors declare that the research was conducted in the absence of any commercial or financial relationships that could be construed as a potential conflict of interest.
